# A case of truncus arteriosus with severe heart failure and pulmonary stenosis: bridge to transplant candidacy with surgical correction and a ventricular-assist device

**DOI:** 10.1007/s10047-024-01456-w

**Published:** 2024-07-15

**Authors:** Kazuki Tanimoto, Takashi Kido, Masaki Taira, Takuji Watanabe, Jun Narita, Hidekazu Ishida, Ryo Ishii, Takayoshi Ueno, Shigeru Miyagawa

**Affiliations:** 1 Department of Cardiovascular Surgery, Osaka University Graduate School of Medicine, 2-15 Yamadaoka, Suita, Osaka 565-0871 Japan; 2 Department of Pediatrics, Osaka University Graduate School of Medicine, 2-15 Yamadaoka, Suita, Osaka 565-0871 Japan

**Keywords:** Ventricular-assist device, Congenital heart disease, Truncus arteriosus, Pulmonary stenosis

## Abstract

Ventricular-assist device therapy for small patients with congenital heart disease is challenging due to its complex anatomy and hemodynamics. We describe a 3-year-old patient with heart failure with truncus arteriosus in the palliative stage. The patient underwent palliative right ventricular outflow tract reconstruction following bilateral pulmonary artery banding. At 6 months of age, the patient developed severe truncal valve regurgitation and left ventricular dysfunction. Emergent truncal valve replacement with a mechanical valve was performed, but left ventricular dysfunction persisted. At 3 years of age, the patient developed acute progression of heart failure triggered by influenza infection. The patient was intubated and transferred to our center to determine the indication for heart transplantation. On the second day after admission, signs of multiorgan failure appeared. Emergent ventricular-assist device implantation for both ventricles was performed with truncal valve closure, ventricular septal defect closure, atrial septal defect closure, and re-right ventricular outflow tract reconstruction. The right ventricular-assist device was successfully removed on the seventh postoperative day. Due to the small pulmonary arteries, severe pulmonary stenosis persisted after ventricular-assist device implantation, but it gradually improved with multiple pulmonary angioplasties. The patient was registered in the Japanese organ transplant network and is awaiting a donor organ in a stable condition.

## Introduction

Following the first pediatric heart transplantation from a donor under 15 years of age in Japan, which was performed at Osaka University in 2011 [1], the number of domestic pediatric heart transplantation procedures has increased. By 2022, a total of 34 patients under the age of 10 years had undergone heart transplantation in Japan.

As the demand for pediatric donor hearts increases, the need for ventricular-assist device (VAD) implantation in small children will also increase. The Berlin Heart EXCOR (BHE) is a VAD that is specifically designed for small children and has been available in Japan since 2015. To date, a total of 117 patients have undergone BHE implantation in Japan. Dilated cardiomyopathy is the most common indication for BHE implantation, followed by congenital heart disease (CHD). However, the outcomes of BHE implantation for CHD have been reported to be worse than for other etiologies [2]. Given the variable anatomical and hemodynamic features of patients with CHD, the indication for BHE implantation should be carefully determined according to each patient’s condition.

Patients with complex CHD often undergo staged repair and may develop heart failure in the palliative phase. In this setting, BHE implantation requires concomitant surgeries, such as atrioventricular valve repair, aortic valve replacement, intracardiac shunt closure, and pulmonary artery (PA) plasty. A small PA at the time of BHE implantation is a matter of particular concern, because PA growth after BHE implantation is uncertain. Moreover, persistent PA stenosis can lead to right ventricular (RV) dysfunction and represents a significant obstacle to heart transplantation.

Herein, we describe a case of a 3-year-old girl with truncus arteriosus with severely reduced left ventricular (LV) function and pulmonary stenosis after palliative RV outflow tract reconstruction (RVOTR) and truncal valve replacement. The patient underwent truncal valve closure, ventricular septal defect closure, and re-RVOTR, combined with BHE implantation for bridge-to-transplant candidacy.

## Case report

**Fig.1:**
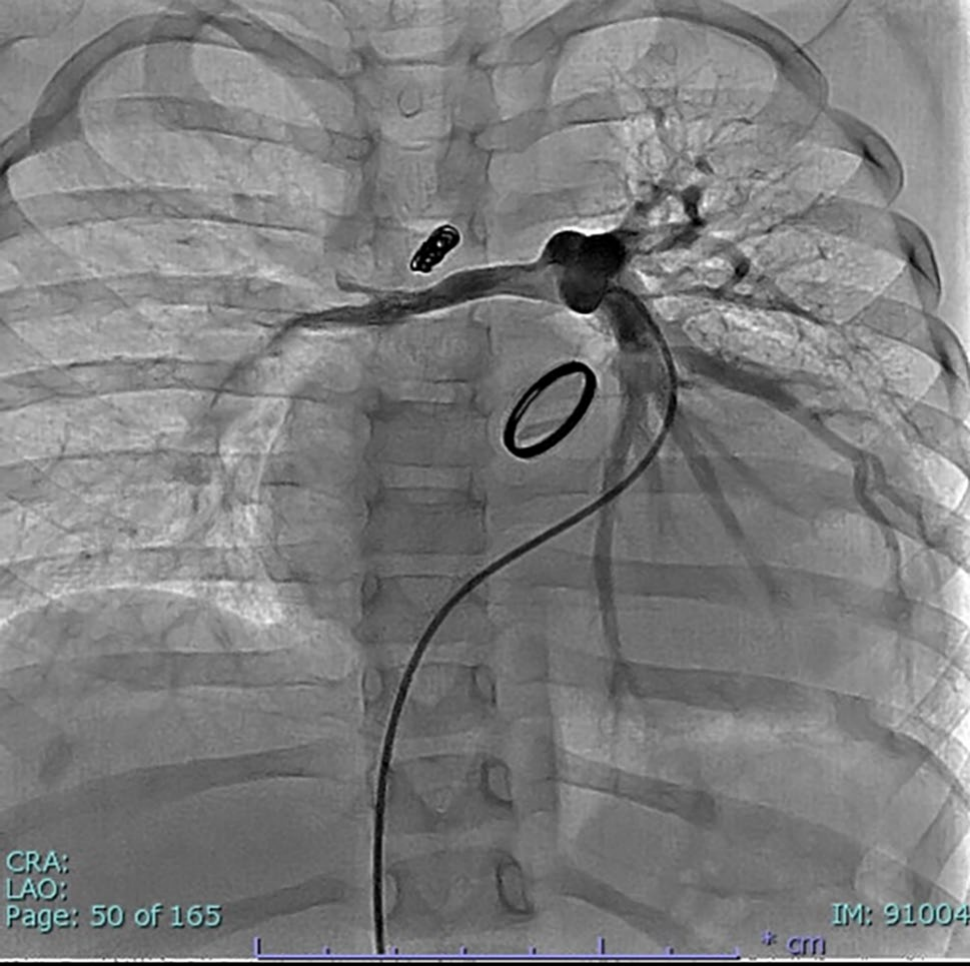
Pulmonary angiogram before transfer to our center

A 3-year-old girl was transferred to our hospital to determine the indication for heart transplantation. The patient was born at 41 weeks of gestation weighing 2.8 kg. Due to respiratory failure, she was admitted to the referring hospital where she was diagnosed with truncus arteriosus type A2. Bilateral PA banding was performed on the 4th day after birth, and the patient was discharged on postoperative day 17. Two months after her birth, palliative RVOTR with the LeCompte maneuver was performed using an 8-mm hand-made polytetrafluoroethylene (PTFE) tri-leaflet valved conduit. The postoperative course was uneventful, and the patient was discharged on postoperative day 13. Six months after her birth, the patient was urgently admitted to the original referring hospital due to progressive deterioration in her general condition. Echocardiogram showed severe truncal valve regurgitation and reduced LV function. Truncal valve replacement with the Konno procedure using the 16 mm ATS Advanced Performance valve (ATS Medical Inc., MN, USA) and atrial septal defect enlargement was emergently performed. Because of the hypoplastic pulmonary arteries and LV dysfunction, ventricular septal defect closure was not performed at this stage. The postoperative course was complicated with atrial arrhythmia and LV dysfunction. The patient was treated with amiodarone and an angiotensin-converting enzyme inhibitor, and she was discharged on postoperative day 83. At 2 years of age, a systemic-to-PA shunt was constructed because of progressive hypoxia. At 3 years of age, the patient developed acute progression of heart failure triggered by influenza type A infection. Considering the possibility of low coronary flow, the systemic-to-PA shunt was embolized with coils. For severe bilateral PA stenosis, balloon dilatations were performed for both PAs. The sizes of the right and left PAs after the procedure were 4.3 mm and 4.5 mm, respectively, and the pressure gradient between peripheral PA and PA trunk was 47 mmHg (Fig. 1). The pressures of right PA, left PA, main PA, and right ventricle were 11/2(7) mmHg, 26/6(17), 47/15(29) mmHg, and 84/edp 7 mmHg, respectively. Despite maximum treatment, the patient’s cardiac function did not recover, and the patient’s parents opted for heart transplantation.

**Fig.2:**
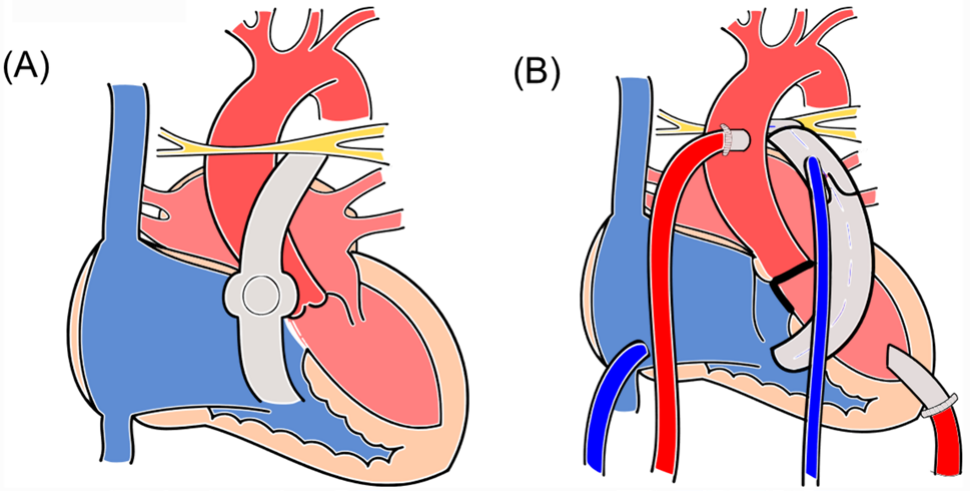
Illustration of the surgery. **A** Before the surgery, **B** after the surgery

**Fig.3:**
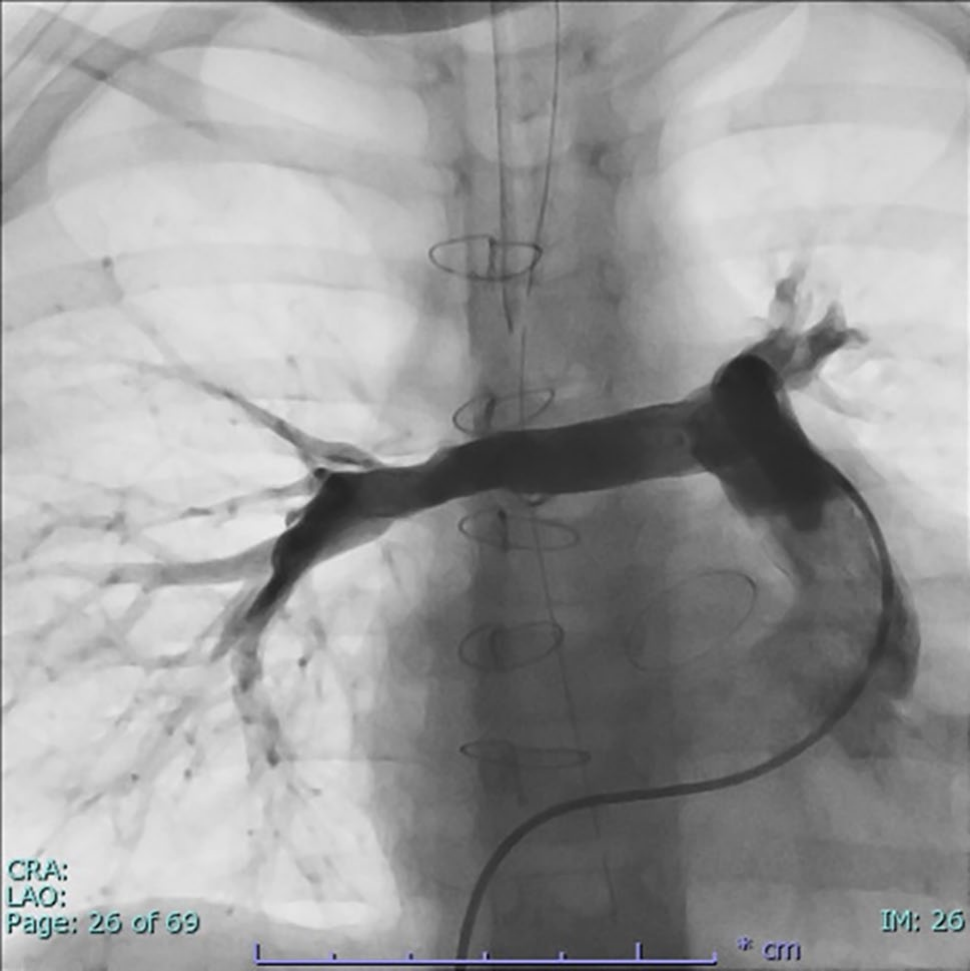
Pulmonary angiogram after Berlin Heart EXCOR implantation

The patient was intubated and transferred to our center by medical jet. On admission, echocardiography showed biventricular dysfunction with an LV ejection fraction of 8% and an RV fractional area change of 28% with inotropic support. Brain natriuretic peptide was markedly elevated to 3131.7 pg/mL. On the 2nd day after admission, signs of multiorgan failure appeared, and emergent biventricular-assist device (BiVAD) implantation was performed. The body size at this surgery was as follows: height of 91 cm, body weight of 10.8 kg, and body surface area of 0.51m^2^. During surgery, truncal valve closure, ventricular septal defect closure, atrial septal defect closure, and re-RVOTR using the 14 mm hand-made PTFE tri-leaflet conduit were concomitantly performed. Regarding truncal valve closure, we removed the mechanical valve (retaining the cuff of it), and then performed a patch closure onto the cuff using a 0.4 mm PTFE patch. Ventricular septal defect closure with 0.4 mm PTFE patch and direct atrial septal defect closure were performed. The central PA was reconstructed behind the ascending aorta using an 8-mm PTFE conduit. Given the impact of residual pulmonary stenosis, a temporary RV-assist device (RVAD) was required. In terms of the VADs, centrifugal pumps were used for both ventricles with an artificial lung in the LVAD circuit. For LVAD cannulae, we chose a 6-mm apex cannula (C18A-020) as inflow cannula and a 6-mm arterial cannula (C19V-020) as outflow cannula. Instead, for RVAD, we inserted 16-Fr inflow cannula (LARGE FLOW^®^ VENOUS RETURN CANNULA) and 10-Fr outflow cannula (Bio-Medicus^®^ NextGen arterial cannula) into IVC via RA and distal pulmonary artery trunk (namely, distal side of the hand-made tri-leaflet valve of the conduit), respectively, both with a purse-string suture. Figure 2 illustrates the surgical procedure. Although postoperative cardiac catheterization showed severe PA stenosis with a pressure gradient of 85 mmHg, biventricular support was effective at stabilizing the patient’s circulation. On postoperative day 7, the RVAD was successfully removed and right PA plasty was performed. On postoperative day 14, the artificial lung was successfully removed. Cardiac catheterization was performed on postoperative day 19, which showed pressure gradients for right and left pulmonary stenosis of 61 mmHg and 83 mmHg, respectively, after PA balloon dilatation. On postoperative day 30, catheter PA balloon dilatation was performed again for pulmonary stenosis. The pressure gradient for right and left pulmonary stenosis decreased to 40 mmHg and 32 mmHg, respectively; namely, the pressures of right PA, left PA, main PA, and right ventricle were 28/15(20) mmHg, 41/16(27), 72/14(37) mmHg, and 78/edp 13 mmHg, respectively. After obtaining in-house approval for heart transplant registration, the LVAD was converted to the BHE (pump size of 15 ml) 35 days after BiVAD implantation. The patient was extubated 3 days after the conversion and discharged from the intensive care unit 5 days after extubation. Cardiac catheterization performed 2 months after intensive care unit discharge showed an improvement in pulmonary stenosis with a pressure gradient of 20 mmHg. The sizes of the right and left PAs were 6.4 mm and 8.6 mm, respectively, and the pressure gradient between peripheral PA and PA trunk was 25 mmHg (Fig. 3). The patient was registered in the Japanese organ transplant network and is waiting for a donor organ in a stable condition.

## Discussion

VAD support for CHD is challenging due to prior cardiac surgery and complex anatomy. Several centers have reported their single-institutional experience of VADs for the functional single ventricle [3, 4]. However, data on VAD therapy for small patients with CHD of the biventricular circulation are limited. Morales et al. demonstrated the outcomes of the BHE in patients with CHD based on the data of all children who underwent BHE implantation in the US between 2007 and 2010 [5]. Forty-one patients had a biventricular physiology, and 49% underwent successful bridge with the BHE. Bleweis et al. recently reported the outcomes of VAD therapy for 44 children with CHD [2]. Among the patients, ten had a biventricular circulation of various etiologies, eight of whom underwent BiVAD implantation, although the detailed outcomes were not described.

The present case of truncus arteriosus presented several problems for bridge-to-transplant candidacy. First, the branch PAs were small and the central PA was constructed anterior to the ascending aorta. Second, the patient was in the palliative stage, and the ventricular septal defect remained patent. Third, the truncal valve was replaced with a mechanical valve. The biggest issue in this case was the severe PA stenosis. The presence of PA stenosis limits the effectiveness of LVAD support due to increased RV pressure load and decreased LV preload. Moreover, persistent PA stenosis may limit the eligibility for heart transplantation. In the present case, surgical PA reconstruction and LVAD implantation with temporary RVAD implantation successfully bridged the patient to transplant candidacy. Although multiple catheter PA plasties were able to promote PA growth, further interventions are still needed to optimize the outcome of heart transplantation for this patient.

Intracardiac shunt is frequently encountered in patients with CHD. To avoid cyanosis, closure of the intracardiac shunt at the time of VAD implantation is recommended based on the recent European Association for Cardio-Thoracic Surgery expert consensus on long-term mechanical circulatory support [6]. Mechanical valves in the aortic position are usually replaced with tissue valves at the time of VAD implantation to prevent thromboembolic events. The patient in the present case underwent truncal valve closure, because the small truncal valve annulus did not allow implantation of a tissue valve of any available size.

Patients with CHD demonstrate a wide spectrum of cardiac anatomical and hemodynamic features. Often, patients with CHD have undergone various types of cardiac surgery, complicating their hemodynamic assessment. Despite these difficulties, previous studies have demonstrated acceptable outcomes with VAD therapy in adult patients with CHD [7, 8]. As shown in these reports, some patients with CHD benefit from VAD therapy. However, a standardized recommendation has not been established due to limited data from small children. For pediatric patients with CHD, each case is discussed individually with a specialized heart team. Increasing the experience of pediatric VAD therapy and improvements in perioperative care have expanded the indications for VAD implantation in small children. Although the present report highlights the difficulties of VAD therapy for small patients with complex CHD and severe PA stenosis, the possibility of applying VADs to these patients needs to be considered.

## Data Availability

The datasets used and/or analyzed during the current study are available from the corresponding author on reasonable request.

## Abbreviations

BiVAD: Biventricular-assist device
BHE: Berlin heart EXCOR
CHD: Congenital heart disease
LV: Left ventricle
LVAD: Left ventricular-assist device
PA: Pulmonary artery
RVAD: Right ventricular-assist device
RVOTR: Right ventricular outflow tract reconstruction
VAD: Ventricular-assist device
PTFE: Polytetrafluoroethylene
